# The efficacy, effectiveness, and safety of Kyung-ok-ko: A narrative review

**DOI:** 10.1097/MD.0000000000031311

**Published:** 2022-11-11

**Authors:** Ji-Woo Kim, Ji-Hye Geum, Won-Bae Ha, Hyeon-Jun Woo, Yun-Hee Han, Shin-Hyeok Park, Jung-Han Lee

**Affiliations:** a Chuna Manual Medicine Research Group, College of Korean Medicine, Won-Kwang University, Iksan, Republic of Korea; b Department of Rehabilitation Medicine of Korean Medicine, Iksan-Jeil Korean Medicine Hospital, Iksan, Republic of Korea; c Department of Korean Medicine Rehabilitation, College of Korean Medicine, Won-Kwang University, Iksan, Republic of Korea; d Department of Acupuncture and Moxibustion Medicine, College of Korean Medicine, Won-Kwang University, Iksan, Republic of Korea; e Research Center of Traditional Korean Medicine, College of Korean Medicine, Won-Kwang University, Iksan, Republic of Korea.

**Keywords:** herbal medicine, herb-drug interactions, Korean traditional medicine, Kyung-ok-ko, narrative review

## Abstract

Kyung-ok-ko (KOK), a traditional medicinal formula in East Asia, has been recently studied across various fields. However, comprehensive reviews of clinical applications of KOK targeting clinical and experimental studies are lacking. Therefore, the application of KOK is being limited to the range of tonic medicines. To overcome this limitation, we aim to investigate the effectiveness, mechanism, and safety of KOK to obtain evidence regarding its effects in clinical applications. We searched for clinical and experimental articles in 11 databases (PubMed, Cochrane Library, Excerpta Medica dataBASE, China National Knowledge Infrastructure, Google Scholar, Research Information Sharing Service, Oriental Medicine Advanced Searching Integrated System, Koreanstudies Information Service System, Korean Medical Database, DBpia, and ScienceON). We selected 54 studies based on the inclusion criteria. Three clinical studies used KOK for a consumptive disease and health promotion. Fifty-one experimental studies reported the antioxidant activity, neuroprotective activity, anticancer effect, anti-inflammatory activity, immunological activity, growth promotion, impacts on cardiovascular system diseases, gastrointestinal system diseases, respiratory system diseases, and metabolic bone disease, hepatoprotective function, and antifatigue function of KOK, which were considered effective and safe in consumptive, chronic, metabolic, inflammatory, and immune diseases. We identified the effectiveness of KOK in the treatment of a wide range of diseases. However, further clinical studies are warranted in the future.

## 1. Introduction

Kyung-ok-ko (KOK), which consists of *Rehmannia glutinosa* var. *purpurea*, *Panax ginseng*, *Poria cocos*, and *Mel*, is a traditional medicinal formula in East Asia.^[1]^ Since its first mention in *Hong-Shi-Ji-Yan-Fang* (洪氏集驗方), it has been recorded in several medical books, such as *Ui-hag-gang-mog* (醫學綱目), *Ui-hag-ib-mun* (醫學入門), and *Dong-ui-bo-gam* (東醫寶鑑). According to a general analysis of medical books, KOK improves health by filling *Jing* (精) and bone marrow, treats dizziness and forgetfulness owing to the lack of brain water, and can be used for a prolonged time in gastrointestinal and respiratory diseases.^[2,3]^

Recent reports have demonstrated the biochemical analysis of KOK and individual herbs consisting of KOK,^[4–10]^ and several experimental and clinical studies related to KOK have been regularly published. In addition, researchers are developing numerous products that use KOK for non-therapeutic purposes, such as food (vinegar,^[11]^ beverages,^[12]^ and yogurt^[13]^) and cosmetics^[14]^; moreover, studies have demonstrated the effectiveness of these products. Therefore, studies are being actively conducted in various fields. However, it is difficult to identify a comprehensive review of the clinical effectiveness of KOK in various diseases, which can be the basis for its use in the clinical field. Furthermore, despite its effects, limited KOK is being used in clinical fields, principally in the range of tonic medicines that improve health.^[3]^ Therefore, we aimed to review clinical and experimental studies related to KOK and analyze their trends and results to address this limitation. In other words, we aimed to present sufficient evidence for the use of KOK in various clinical fields and to suggest directions for future research.

## 2. Methods

### 2.1. Search strategy

This narrative review was designed and performed in 2022 to identify articles on KOK. We used the following 11 databases: PubMed, Cochrane Library, Excerpta Medica dataBASE, China National Knowledge Infrastructure, Google Scholar for other countries, and 6 Korean databases (Research Information Sharing Service, Oriental Medicine Advanced Searching Integrated System, Koreanstudies Information Service System, Korean Medical Database, DBpia, and ScienceON). Table 1 presents the search terms for each database.

**Table 1:** Electric database and search terms used for this study.

Electric databases	Domain	Search terms
RISS	http://www.riss.kr	경옥고
OASIS	http://oasis.kiom.re.kr	
KISS	https://kiss.kstudy.com	
KMbase	https://kmbase.medric.or.kr	
DBpia	https://www.dbpia.co.kr	
ScienceON	https://scienceon.kisti.re.kr	
PubMed	https://pubmed.ncbi.nlm.nih.gov	(Kyungohkgo) OR (Kyungokgo) OR (KyungOkKo) OR (Kyeongokgo) OR (KyungOcGo) OR (Gyungokgo) OR (瓊玉膏) OR (琼玉膏) OR (QiongYugao)
Cochrane library	https://www.cochranelibrary.com	Kyungohkgo in Title Abstract Keyword OR Kyungokgo in Title Abstract Keyword OR KyungOkKo in Title Abstract Keyword OR Kyeongokgo in Title Abstract Keyword OR KyungOcGo in Title Abstract Keyword Gyungokgo in Title Abstract Keyword OR 瓊玉膏 in Title Abstract Keyword OR 琼玉膏 in Title Abstract Keyword OR QiongYugao in Title Abstract Keyword
EMBASE	https://www.embase.com	kyungohkgo:ti,ab,kw OR kyungokgo:ti,ab,kw OR kyungokko:ti,ab,kw OR kyeongokgo:ti,ab,kw OR kyungocgo:ti,ab,kw OR gyungokgo:ti,ab,kw OR 瓊玉膏:ti,ab,kw OR 琼玉膏:ti,ab,kw OR qiongyugao:ti,ab,kw
CNKI	https://www.cnki.net	(Title, Keyword and Abstract: 瓊玉膏(精确)) OR (Title, Keyword and Abstract: 琼玉膏(精确)) OR (Title, Keyword and Abstract: QiongYugao(精确))
Google Scholar	https://scholar.google.co.kr	allintitle: Kyungohkgo OR Kyungokgo OR KyungOkKo OR Kyeongokgo OR KyungOcGo OR Gyungokgo OR 瓊玉膏 OR 琼玉膏 OR QiongYugao

경옥고 = Korean word of Kyung-ok-ko.

### 2.2. Eligibility criteria

The search was conducted from April 12, 2022 to April 19, 2022, and was limited to studies published until December 31, 2021. First, we excluded theses and dissertations from the search. Other specific inclusion and exclusion criteria for the selection of studies were set as follows.

#### 2.2.1. Inclusion criteria.

1. Experimental studies (in vitro, in vivo, and ex vivo studies).
2. All clinical studies targeting humans without limiting the patient’s age, sex, period, and study design.
3. Studies published in Korean, English, and Chinese.

#### 2.2.2. Exclusion criteria.

1. Studies not published in journals.
2. Studies with unavailable original text.
3. Literature review.
4. Grey publications, such as protocols, dissertations, and posters.
5. Studies in which the effect of KOK is not the primary topic.
6. Studies comparing the effects of each KOK manufacturing method.
7. Studies analyzing commercialized KOK rather than therapeutic use.

### 2.3. Data collection and extraction

**Figure 1.**
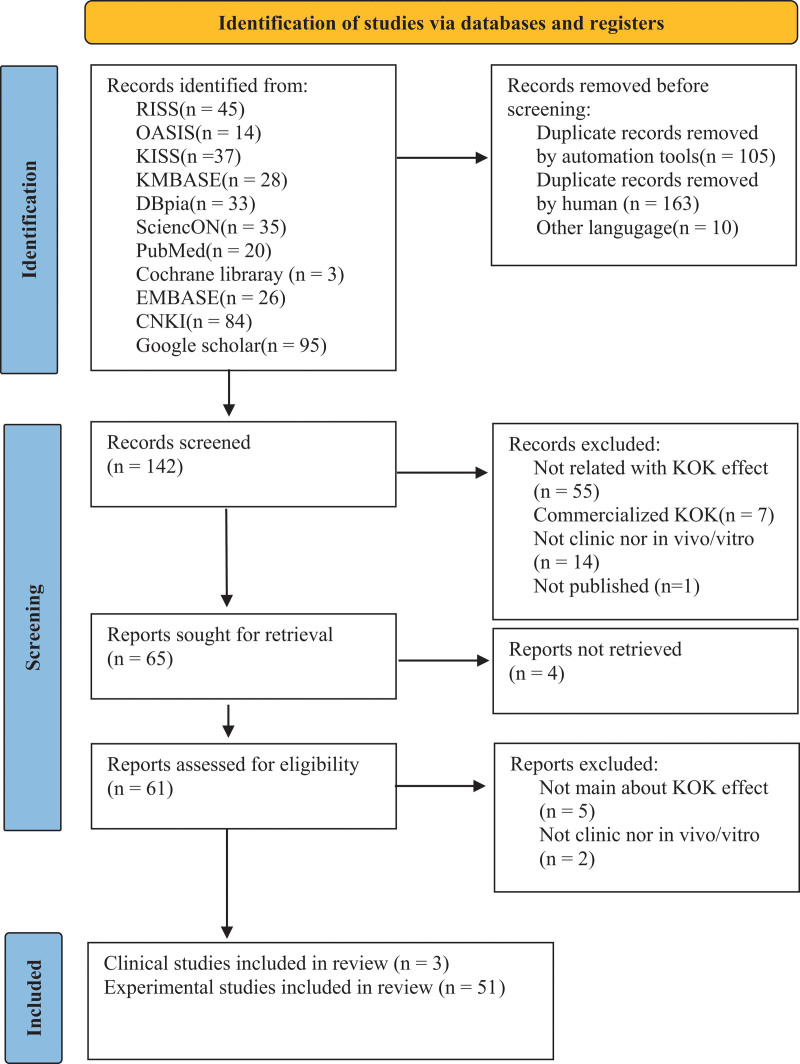
PRISMA flow chart for the database search in this study. KOK = Kyung-ok-ko, PRISMA = Preferred Reporting Items of Systematic Reviews and Meta-Analyses.

A flowchart of the study selection process is shown in Figure 1. First, we conducted a search based on the search strategy and identified 420 articles. Screening was conducted according to the selection criteria based on 142 studies, following the removal of articles not published in Korean, English, or Chinese and duplicates. The first screening was conducted using the title and abstract, whereas the second screening involved reviewing the original text. Two authors (Ji-Woo Kim and Ji-Hye Geum) reviewed the literature independently. They discussed differences in the process and results of the literature evaluation or adjusted their opinions through discussion with a third author (Jung-Han Lee).

## 3. Results

### 3.1. The selection of target research

A total of 54 studies were eventually selected, which comprised 3 clinical studies and 51 experimental studies.

### 3.2. Clinical study review

For the selected clinical studies, we analyzed the results of the study design, disease, composition, and administration methods (Table 2).

**Table 2:** Main data of clinical studies.

References	Study design	Sample size	Conditions	Other treatment	Composition of KOK	Main outcome
Yin et al^[15]^	RCT	60 (30/group)	Tuberculosis (Pulmonary-Yin-deficiency)	W-med	*	1) Effective rate: CG 80.00%, e.g. 93.33% 2) Th1↑, Th2↓
Kim et al^[16]^	RCT	24 (12/group)	Normal soccer player	-	*	1) Aerobic exercise capacity: IMP 2) Fatigue recovery: IMP
Wang^[17]^	Case report	16	Weakness from long-term illness (qi-deficiency)	-	*, LR, AR, LRC	1) Symptoms: IMP 2) Infection resistance: IMP (prevalence rate↓) 3) No side effect

* = Original composition of KOK, - = not reported, AR = *Asparagi radix*, CG = control group, EG = experimental group, IMP = improved, KOK = Kyung-ok-ko, LR = *Liriopes radix*, LRC = *Lycii radicis cortex*, RCT = randomized controlled trial, Th = T helper cell, W-med = Western medicine.

#### 3.2.1. Study design analysis.

The clinical studies consisted of 2 randomized controlled trials^[15,16]^ and 1 case report.^[17]^

#### 3.2.2. An analysis of the KOK composition and medication method.

Two studies used the original composition of KOK,^[15,16]^ whereas 1 study administered KOK by adding *Liriope platyphylla*, *Asparagus cochinchinensis*, and *Lycium chinense*.^[17]^

Regarding the dosing frequency, all 3 studies were based on twice-a-day dosing.^[15–17]^ A total of 4 weeks of duration was mentioned in 2 studies,^[15,16]^ but not in 1 study.^[17]^

#### 3.2.3. Combined treatment analysis.

One study mentioned combined treatment; the similar Western medicine administered to the control group was administered in combination with KOK to the experimental group, and its efficacy was analyzed.^[15]^ No combined treatment was performed in the remaining 2 studies. Each of them analyzed the efficacy of KOK compared with placebo,^[16]^ and the efficacy of KOK alone.^[17]^

#### 3.2.4. Applied disease.

One study used KOK based on its diagnosis name, which was used for pulmonary tuberculosis,^[15]^ whereas the remaining 2 studies used KOK according to patient complaints.^[16,17]^ Specifically, they reported on pulmonary tuberculosis, which is a patternized deficiency of pulmonary-*Yin*,^[15]^ fatigue in normal athletes,^[16]^ and weakness following a prolonged illness, which refers to patternized *Qi*-deficiency.^[17]^

#### 3.2.5. Result analysis.

Two studies reported the improvement of subjective symptoms in patients, and each demonstrated significant improvements in the relief of symptoms, such as coughing, sputum, hectic fever, and night sweats related to pulmonary tuberculosis,^[15]^recovery from poor appetite, and physical strength related to weakness.^[17]^

Two studies reported improvements in the objective indicators. Specifically, they demonstrated a decrease in calcification on pulmonary imaging, *Mycobacterium tuberculosis* test negativity, increased T helper cells (Th)1, and decreased Th2 in diagnostic laboratory tests as the indicators of pulmonary tuberculosis.^[15]^ Moreover, an improvement in maximum oxygen consumption volume and a reduction in the heart rate were reported as the indicators of fatigue.^[16]^

#### 3.2.6. Side effect.

One study mentioned that side effects did not appear during treatment.^[17]^ No side effects have been reported in other studies.

### 3.3. Experimental study review

Table 3 summarizes the analysis of the study design, composition, and primary results of the selected experimental studies.

**Table 3:** Main data of experimental studies. The name of medicinal herbs was written in Latin including used parts.

References	Study design	Composition of KOK	Activity/main mechanism
Xue and Au^[18]^	In vivo	*	Delay aging/antioxidant
Kwak et al^[19]^	In vivo	*	Delay aging/antioxidant
Xue and Li^[20]^	In vivo	*	Improvement of the central nervous system/antioxidant of hypothalamus and delay brain neuronal disturbance
Qian and Wei^[21]^	In vivo	*	Anti-aging skin/antioxidant and hydroxyproline, hyaluronic acid, vascular endothelial growth factor, and basic fibroblast growth factor↑
Qu et al^[22]^	In vivo	*	Delay aging/antioxidant and anti-inflammatory
Liu et al^[23]^	In vivo	*	Delay aging/antioxidant
Hwang et al^[24]^	In vivo	*, LF, ARL, ss	Spermatogenic ability/antioxidant and Inhibiting apoptosis
Jo and Choi^[25]^	In vitro	-	Inflammation of atopic dermatitis↓/antioxidant and anti-inflammatory
Liu et al^[26]^	In vitro and in vivo	RR, P, KRPG, M	Anti-depressive/anti-inflammatory and antioxidant
Lee and Bae^[27]^	In vitro and in vivo	*, LF, ARL, ss	Inhibited particulate matter-induced vascular barrier disruptive responses/antioxidant and anti-inflammatory
Choi et al^[28]^	In vivo	*, LF, ARL, ss	Mitigate neurotoxicity and anti-blood-brain-barrier disruption/antioxidant and anti-inflammatory
Cai et al^[29]^	In vivo	*, LF, ARL, ss	Neuroprotective and attenuation of memory impairment/anti-inflammatory
Shin et al^[30]^	In vitro and in vivo	*, LF, ARL, ss	Memory ameliorating/inhibit acetylcholinesterase activity
Park et al^[31]^	In vivo	1) RR, P, GR 2) RR, P, GR, AGR, GR, HH, OCR, LgR, SR, WG	Learning ability and memory improver
Cho et al^[32]^	In vivo	*	Amelioration and prevention of cognitive deficits and depression among menopausal symptoms/mature brain-derived neurotrophic factor (mBDNF)↑
Liu et al^[33]^	In vivo	*	Anti-aging/down-regulation of acetonic and amino acid metabolism
Liu et al^[34]^	In vivo	*	Delay aging/selection of candidate target proteins for delaying aging
Zhang et al^[35]^	In vitro and in vivo	*	Delay aging/anti-inflammatory
Zhang et al^[36]^	In vitro	*	Enhance the effects of anti-lung cancer chemotherapy(cisplatin)/-
Chen and Shen^[37]^	In vivo	*	Enhance the effects of anti-lung cancer chemotherapy(cisplatin)/nucleoside diphosphate kinase A (nm23)↑ and Proliferating cell nuclear antigen↓
Chen and Shen^[38]^	In vitro	*	Enhance the inhibition effects of anti-lung cancer chemotherapy(cisplatin)/G1 period↑ and S period↓ of cancer cells
Chen et al^[39]^	In vivo	*	Enhance the attenuation effects of anti-lung cancer chemotherapy(cisplatin)/-
Lee et al^[40]^	In vivo	1) * 2) RR, P, RPG, M, LE, CV	Anti-lung cancer/-
Liu et al^[41]^	In vivo	*	Attenuate pancreatic cancer with chemotherapy(gemcitabine)/Th1/Th2 and Th/Treg ratio↓, white blood cell↑, and Red blood cell↑
Chen et al^[42]^	In vivo	*	Prophylaxis and treatment of hepatocellular carcinoma/inhibited hepatitis B virus X antigen expression
Chen et al^[43]^	In vivo	*	Improve bone marrow inhibition induced by cancer chemotherapy(cisplatin)/erythrocyte, leucocyte, and blood platelets in bone marrow↑
Chen^[44]^	In vivo	*	Alleviating immune suppression induced by cancer chemotherapy(cisplatin)/IL-2 and TNF↑
Chen^[45]^	In vitro and in vivo	*	Improve bone marrow inhibition induced by cisplatin/bone marrow nuclear cells↑, increasing S period, and decreasing G1 period of cells
Teng et al^[46]^	In vitro and in vivo	RR, P, GR	Protects against cisplatin-induced nephrotoxicity without reducing anti-tumor activity/anti-inflammatory and reducing platinum accumulation in the kidney
Jang et al^[47]^	In vivo	*, LF, ARL, ss	Alleviates polycystic ovarian syndrome/regulate immunological pathway and anti-inflammatory
Lee et al^[48]^	In vitro and in vivo	*, LF, ARL, ss	Suppressing polycystic ovarian syndrome/regulate immunological pathway and anti-inflammatory
Roh et al^[49]^	In vivo	*	Enhance immunity/Th1, interferon gamma, IL-2, IL-12, Th2, IL-4, IL-5, IL-13, spleen cells, T cells, B cells, and macrophages↑
Lee et al^[50]^	In vitro	1) * 2) RR, P, RPG, M, LE, CV	Activate immunity/the ability of macrophages to produce nitric oxide and TNF-a↑
Im et al^[51]^	In vivo	*	Inhibition of atopic dermatitis/downregulated IgE and reduced the infiltration of mast cell
Do et al^[52]^	In vivo	*, PMR	Hair growth/IGF-1 and vascular endothelial growth factor↑
Cha et al^[53]^	In vivo	*, CP	Growth effect/red blood cell, hemoglobin, and hematocrit↑
Han et al^[54]^	In vivo	*	Growth and learning effect/red blood cell and hematocrit↑
Jung et al^[55]^	In vivo	*, CH, MO, CR, CP	Growth effect/IGF-1 and thyroid stimulating hormone (TSH)↑
Kim et al^[56]^	Ex and in vivo	*, LF, ARL, ss	Anti-platelet and anti-thrombotic/adenosine triphosphate (ATP) release↓, intracellular Ca2+↑, and the phosphorylation of phospholipase C gamma and protein kinase B
Kim and Song^[57]^	In vivo	1) * 2) *, SM 3) *, SM, CF, AtR, CS, HF	Anti-hyperlipidemia/total cholesterol and triglyceride↓ and high density lipoprotein-cholesterol↑
Whang et al^[58]^	In vivo	*	Antihyperlipidemia, antihypertension, antifatigue, and weight loss/cholesterol and triglyceride↓
Shin and Yang^[59]^	In vitro	*	Oxidative damage defense of myocardial cell/Heme oxygenase-1↑, Fas↓, Fas ligand↓, and Bcl-XS↓
Wen^[60]^	In vivo	*	Laxation/-
Chen et al^[61]^	In vivo	*	Protect gastric mucosa/gastric mucositis cells and neutrophils↓
Whang et al^[62]^	In vivo	*	Anti-inflammatory and alleviates gastric ulcer and pain and regulate temperature/-
Hu et al^[63]^	In vivo	*	Anti-inflammatory, expectorant, and antitussive/-
Jeon et al^[64]^	In vitro	*	Decrease antituberculosis drug dosage and drug resistance/-
Hwang et al^[65]^	In vitro and in vivo	1) * 2) *, HD	Antiosteoporosis/lower serum calcium, phosphorus, alkaline phosphatase, CD4+, CD8+, and CD11c + cells↓ and serum estradiol↑
Kim et al^[66]^	In vitro and in vivo	1) * 2) *, NS	Inhibit osteoclast and increase osteoblasts/-
Kwon and Kim^[67]^	In vivo	*	Improve and prevent liver injury/GOT and GPT↓
Kim et al^[68]^	In vivo	*	Anti-fatigue/serum lactate↓, serum glucose↑, and glycogen in muscle↑

* = Original composition of KOK, - = Not reported, ↑ = Increase, ↓ = Decrease, AGR = *Angelicae gigantis radix*, AtR = *Astragali radix*, ARL = *Aquilariae resinatum lignum*, CD = cluster of differentiation, CF = *Crataegii fruc*tus, CH = *Cistanches herba*, CP = *Cervi pantotrichum cornu*, CR = *Cibotii rhizoma*, CS = *Cassiae semen*, CV = *Cordyceptis vermis*, GOT = glutamic oxaloacetic transaminase, GPT = glutamic pyruvate transaminase, GR = *Ginseng radix*, HD = *Hovenia dulcis*, HF = *Hordei fructus germiniatus*, HH = *Hydrangeae herba*, Ig = immunoglobulin, IGF = Insulin-like growth factor, IL = interleukin, JGR = Japanese *angelicae radix*, KRPG = Korean red *panax ginseng*, LE = *Lentinula edodes*, LF = *Lycii fructus*, LgR = *Ligustici rhizoma*, M = *Mel*, MO = *Morindae radix*, NS = N*elumbo semen*, OCR = *Opuntiae caulis et radix*, P = *Poria*, PMR = *Polygoni multiflori radix*, RR = *Rehmanniae radix*, RPG = Red red *panax ginseng*, SR = *Scutellariae radix*, ss = Simple syrup, SM = *Salviae miltiorrhizae radix*, Th = T helper cells, TNF = tumor necrosis factor, WG = Wild-simulated *ginseng*.

#### 3.3.1. Study design analysis.

Of the 51 experimental studies, 35, 6, 9, and 1 were in vivo, in vitro, in vivo and in vitro, and ex vivo and in vivo studies, respectively. All in vivo studies used mice as the test subjects, whereas *Drosophila melanogaster* was used as a test subject in the study by Xue et al.^[18]^

#### 3.3.2. The composition of KOK.

Of all the experimental studies, 33 studies used the original formulation composed of *Rehmannia glutinosa* var. *purpurea*, *Panax ginseng*, *Poria cocos*, and *Mel*, whereas 18 studies added other herbs to the original formulation. *Lycium chinense* and *Aquilaria agallocha* were the most commonly added and were mentioned in 8 studies. Three studies used red ginseng instead of *Panax ginseng*, and 2 studies each added *Cervus nippon*, *Lentinula edodes*, and *Cordyceps sinensis*. *Angelica sinensis, Hydrangea macrophylla*, *Opuntia ficus-indica*, *Ligusticum chuanxiong*, *Scutellaria baicalensis*, wild-simulated ginseng, *Polygonum multiflorum*, *Cistanche salsa*, *Morinda officinalis*, *Cibotium barometz*, *Salvia miltiorrhiza*, *Crataegus pinnatifida*, *Astragalus membranaceus*, *Cassia tora*, *Hordeum vulgare*, *Hovenia dulcis*, and *Nelumbo nucifera* were added to KOK.

#### 3.3.3. Result analysis.

**Figure 2.**
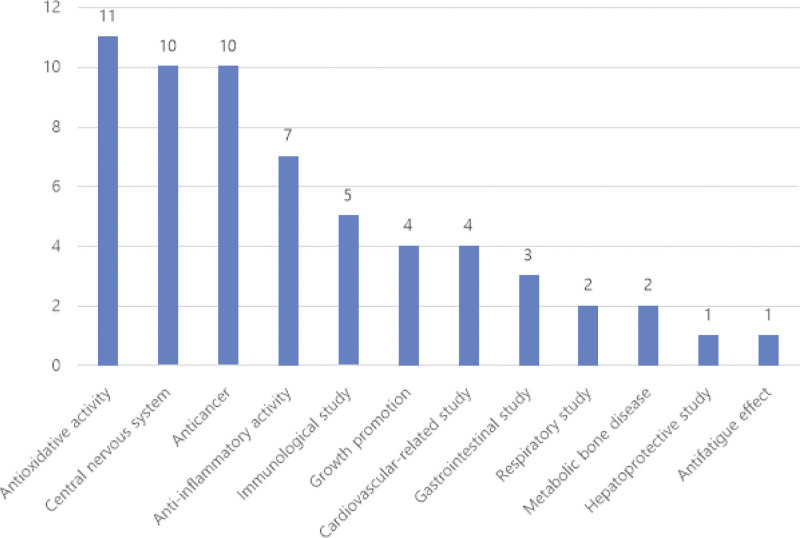
The number of cases per effect in experimental studies. The numbers have been counted as plural.

The antioxidative activity analyzed in the 11 experimental studies demonstrated the most significant effect. In addition, the central nervous system and cancer had 10 reported studies each. Moreover, the studies reported on anti-inflammatory, immunological, growth-related, cardiovascular, gastrointestinal, respiratory, metabolic bone disease, hepatoprotective, and antifatigue effects (Fig. 2).

##### 3.3.3.1. Antioxidative activity

Eleven studies have demonstrated the mechanisms underlying the antioxidative activity of KOK. Superoxide dismutase^[18–23]^ and glutathione peroxidase^[18–20,22–24]^ showed the highest frequency of activation via antioxidative mechanisms, with 6 studies each. Three studies reported on antioxidative mechanisms through the reduction of reactive oxygen species (ROS).^[25–27]^ Moreover, researchers have demonstrated various antioxidative mechanisms, such as the inhibition of nitric oxide (NO) and inducible NO,^[26]^ the reduction of plasma thiobarbituric acid reactive substance,^[19]^ lipid peroxidation,^[20]^ malondialdehyde,^[21]^ and the activation of the anti-Kelch-like ECH-associated protein-anti-nuclear factor erythroid 2-related factor2 pathway.^[28]^

Regarding the efficacy of KOK through antioxidant mechanisms, 6 studies reported on anti-aging efficacy,^[18–23]^ whereas the remaining studies demonstrated relief from inflammation in atopic dermatitis,^[25]^ relief from depression,^[26]^ increased sperm production,^[24]^ relief from neurotoxicity,^[28]^ and suppression of vascular barrier destruction.^[27]^

##### 3.3.3.2. Central nervous system

Researchers have performed 10 studies on KOK and the central nervous system. Specifically, relief from memory impairment^[29–32]^ displayed the highest frequency, as reported in 4 studies. In addition, it could be divided into 3, 2 each, and 1 study on anti-aging,^[33–35]^ depression improvement^[26,32]^ and recovery from nervous system damage,^[20,28]^ and neuroprotection, respectively.^[29]^

In a detailed analysis of the studies analyzing the mechanisms which most significantly impact the central nervous system, anti-inflammatory activity^[28,29,35]^ and antioxidant activity^[20,26,28]^ were the most commonly investigated, each being evaluated in 3 studies. Furthermore, the corresponding effect was analyzed by inhibiting acetylcholinesterase,^[30]^ regulating the metabolism of acetonic acid and amino acids,^[33]^ and affecting the brain target proteins.^[34]^

##### 3.3.3.3. Anticancer effect

Ten studies reported anticancer effects, of which 7 studies reported a direct anticancer effect,^[36–42]^ and 3 studies mentioned the alleviating side effects of cisplatin chemotherapy during cancer treatment.^[43–45]^ Specifically, lung cancer was reported in 8 studies,^[36–40,43–45]^ whereas pancreatic cancer^[41]^ and liver cancer^[42]^ were each reported in 1 study.

Three studies reported alleviation of the side effects of chemotherapy following lung cancer, which were associated with the suppression of bone marrow^[43,45]^ and relief from immunosuppression.^[44]^

##### 3.3.3.4. Anti-inflammatory activity

Seven studies reported the anti-inflammatory activity of KOK. Specifically, some studies have reported anti-inflammatory activity through the reduction of cytokines^[27]^ (interleukin [IL]-1β,^[28,29,46–48]^ tumor necrosis factor-α,^[35,46,47]^ and IL-6^[28,47,48]^), chemokines (IL-8 and monocyte chemoattractant protein-1),^[47,48]^ cyclooxygenase-2,^[28,46]^ and inducible NO.^[28,47]^ Moreover, anti-inflammatory activity is activated through the inhibition of the nuclear factor-kappa B signaling pathway^[28,35]^ and mitogen-activated protein kinases.^[28]^

##### 3.3.3.5. Immunological study

Five studies demonstrated immune function. First, a study improved immunity by alleviating the decrease in spleen cells, T cells, B cells, and macrophages, which are the immunotoxic effects of methotrexate, while restoring Th1 and Th2 imbalance^[49]^ Another study increased immune activity through the activation of macrophages.^[50]^ In addition, 1 study reported a beneficial effect^[51]^ on atopic dermatitis through the reduction of immunoglobulin E, whereas 2 studies suppressed polycystic ovarian syndrome through the reduction of cluster of differentiation 8 and macrophages along with anti-inflammatory activity.^[47,48]^

##### 3.3.3.6. Growth promotion

Four studies have reported growth promotion, of which 1 demonstrated hair growth and the remaining demonstrated physical growth. The expression of proteins related to hair growth factors, such as insulin-like growth factor-1 and vascular endothelial growth factor, promoted hair growth during hair loss.^[52]^ Regarding physical growth, 2 studies reported on the promotion of growth by increasing the total number of red blood cells and packed cell volume in growth disorders owing to nutrient deficiency.^[53,54]^ By contrast, another study confirmed that increasing insulin-like growth factor-1 and thyroid-stimulating hormone exerted a significant growth effect.^[55]^

##### 3.3.3.7. Cardiovascular study

Four of the studies were related to the cardiovascular system. Specifically, they reported anti-thrombotic activity,^[56]^ anti-hyperlipidemic effects,^[57,58]^ and protection against oxidative damage to cardiomyocytes.^[59]^

##### 3.3.3.8. Gastrointestinal study

Three studies were related to the gastrointestinal system, and each study reported the significant effects of KOK on laxation,^[60]^ the protection of gastric mucosa,^[61]^ acute and chronic anti-inflammatory effects, ulcer suppression, and analgesia.^[62]^

##### 3.3.3.9. Respiratory study

Two studies were related to the respiratory system. One study reported expectorant and antitussive effects,^[63]^ whereas another demonstrated the efficacy of KOK in antituberculosis and reduced drug resistance when co-administered with antituberculosis agents.^[64]^

##### 3.3.3.10. Metabolic bone disease

Two studies were related to metabolic bone diseases. One study reported on a significant effect on osteoporosis owing to estrogen deficiency,^[65]^ whereas another reported on the inhibition of osteoblast proliferation and bone resorption in inflammatory bone loss.^[66]^

##### 3.3.3.11. Hepatoprotective study

One study reported the hepatoprotective effect of KOK, which demonstrated the improvement and prevention of liver damage by not only inhibiting serum glutamic oxaloacetic transaminase and glutamic pyruvate transaminase activation, but also suppressing the formation of thiobarbituric acid and improving lesions of hepatic tissues in combination with glutathione.^[67]^

##### 3.3.3.12. Antifatigue effect

One study reported the antifatigue effect of KOK by decreasing serum lactate and increasing serum glucose and intramuscular glycogen levels.^[68]^

##### 3.3.3.13. Toxicity and side effects of KOK

Eight studies reported the toxicity and side effects of KOK. Of them, 6 studies conducted experiments on the toxicity of KOK and predominantly demonstrated the absence of cytotoxicity.^[25–27,47,56,65]^ Specifically, 1 study reported that average weight, aspartate aminotransferase, alanine aminotransferase, lactic dehydrogenase, tumor necrosis factor-a, and Fas in the liver and kidney were unaffected by KOK administration.^[47]^ Also, KOK did not induce toxicity even when administered for a long time.^[47]^

Three studies mentioned the side effects of KOK. Some studies reported that serum glutamic oxaloacetic transaminase and glutamic pyruvate transaminase levels did not change during KOK administration; therefore, KOK did not exert a negative effect on liver function.^[42,53]^ Regarding the anti-thrombotic effect of KOK, 1 study reported its potential advantage over aspirin in terms of side effects due to shorter bleeding time compared with aspirin.^[56]^

## 4. Discussion

KOK has been described in various medical books, such as *Ui-hag-gang-mog* (醫學綱目), *Ui-hag-ib-mun* (醫學入門), and *Dong-ui-bo-gam* (東醫寶鑑), since its first report in *Hong-Shi-Ji-Yan-Fang* (洪氏集驗方). Moreover, it is 1 of the most widely used prescriptions in East Asia consisting of *Rehmannia glutinosa* var. purpurea, *Panax ginseng*, *Poria cocos*, and *Mel*.^[1,2]^ It can be used to improve health by filling *Jing* (精) and bone marrow or for consumable cold and *Jo* (燥) patterns because of body fluid deficiency.^[1]^ Particularly, *Ui-hag-ib-mun*, *Jingyue Quanshu* (景岳全書), and *Dong-ui-bo-gam* mentioned that 1 to 3 herbs could be added to the original composition of KOK to produce *Gami*-KOK with specific effects.^[2]^ KOK can be generally used for tonic medicine and consumptive diseases, and can also be used for various diseases by appropriately adding or subtracting herbs.^[2,3]^

Researchers have actively investigated and reported the biochemical analysis of KOK and its individual herbs consisting of KOK in recent years. KOK contains amino acids (valine, aspartic acid, and arginine) and 14 minerals (K and Na).^[4]^ Particularly, the extract using chloroform as a solvent displayed highest antioxidative activity.^[4]^ Moreover, while examining each of the studies on the individual herbs that constitute KOK, the primary herb *Rehmannia glutinosa* var. *Purpurea* displays efficacy in various inflammatory and metabolic diseases owing to its anti-inflammatory, antioxidant, hypoglycemic, and autonomic nervous system activities.^[5]^ *Poria cocos* is composed of chemical components, such as triterpenes and polysaccharides, which effectively inhibit cytokine secretion, enhance immunity, anticancer, and gastrointestinal and renal diseases.^[6,7]^ *Panax ginseng* comprises ginsenoside as the major component, and displays antioxidant, anti-inflammatory, and immune-stimulating activities by inhibiting the production of ROS and promoting NO production.^[8–10]^ Moreover, it is effective in the cardiovascular system, neurodegenerative diseases, diabetes, and complications owing to these activities.^[8–10]^ Furthermore, numerous experimental and clinical studies have demonstrated the effects of KOK; however, it is difficult to identify comprehensive research. One review study on the efficacy of KOK based on historical medical books exists, however, it focused on medical books and analyzed only 7 Korean studies.^[3]^ These reasons eventually contributed to narrowing the range of KOK use in clinical fields. Currently, KOK is principally used in a limited range of tonic medicines.^[3]^ Therefore, we reviewed and analyzed KOK-related studies through 11 database searches to collect sufficient evidence for its clinical use and suggest directions for future research.

A total of 54 studies were related to KOK, of which 3 were clinical studies.^[15–17]^ Each study focused on pulmonary tuberculosis, a patternized deficiency of pulmonary-*Yin*,^[15]^ fatigue,^[16]^ and weakness following prolonged illness and patternized *qi*-deficiency.^[17]^ They reported on the useful effects of KOK as a treatment for diseases as well as a tonic medicine. In addition, 1 study by Wang^[17]^ used a combination of *Liriope platyphylla*, *Asparagus cochinchinensis*, and *Lycium chinense* with KOK, which were added from *Dong-ui-bo-gam*. This can form the basis for the increase in the range of KOK through the addition of the aforementioned herbs. Unlike these studies, 1 study combined KOK with Western medicine; nonetheless, a control group was treated only with identical Western medicine.^[15]^ Eventually, all clinical studies revealed a single effect of KOK.

We identified 51 experimental studies on KOK. In addition to the original composition of KOK, various herbs were added to it. Specifically, 33 studies used the original composition, whereas 18 studies added herbs. *Lycium chinense* and *Aquilaria agallocha* were the most commonly added herbs, and were identified in 8 studies. *Lycium chinense* inhibits malondialdehyde formation, activates the removal of superoxide anions and anti-superoxide formation,^[69]^ prevents or alleviates oxidative stress-induced hepatotoxicity.^[70]^ Furthermore, it is useful as a treatment for learning and memory deficits induced by trimethyltin.^[71]^ *Aquilaria agallocha*, with 4-butyl-a-agarofuran as the primary component, exerted an anxiolytic effect in an animal model.^[72]^ Based on the efficacy of these herbs, their combination with KOK can enhance its antioxidative activity and effect on the central nervous system.

Red ginseng was used instead of *Panax ginseng* in 3 studies, and was principally used to relieve depression,^[26]^ anticancer effects,^[40]^ and immune activity.^[50]^ Only 1 study used red ginseng instead of *Panax ginseng*, without the addition of other herbs, and it was reportedly effective in relieving depression.^[26]^ This result was consistent with that of another study demonstrating that red ginseng alleviates depression by improving the function of the astrocytic gap junction.^[73]^ Therefore, red ginseng can be used instead of *Panax ginseng* in the original composition upon the use of KOK in patients with depression.

In contrast, studies that added other herbs had a limitation in that it is difficult to analyze the efficacy of KOK itself. Thus, we identified studies that compared the efficacies of KOK and *Gami*-KOK, which suggested that some herbs were added to KOK. KOK itself was mentioned to display the expected efficacy; nonetheless, it can produce better effects upon using *Gami*-KOK.^[53,57,66]^ This finding was consistent with the mention in the *Ui-hag-ib-mun*, *Jingyue Quanshu*, and *Dong-ui-bo-gam* that *Gami*-KOK can be manufactured with a specific function by adding 1 to 3 herbs to KOK. Therefore, upon performing additional complementary research in the future, KOK will likely treat various diseases by adding herbs based on the symptoms of a patient in actual clinical practice.

Regarding the effects of KOK in experimental studies, 11, 10 each, 7, 5, 4, 3, 2 each, and 1 each study demonstrated significant antioxidative activity, diseases of the central nervous system and anticancer effect, anti-inflammatory activity, immune activity, growth promotion and cardiovascular system diseases, diseases of the gastrointestinal system, respiratory system diseases and metabolic bone diseases, and hepatoprotective and antifatigue effects, respectively. The predominantly analyzed antioxidative activity, which refers to the removal of free radicals generated in the body, has attracted interest in modern medicine.^[74,75]^ This is because oxidative stress owing to an increase in free radicals changes the oxidation-reduction state of cells and induces inflammation.^[76]^ These harmful actions affect the liver, central nervous system, heart, and testicles, thus causing chronic diseases and metabolic disorders.^[74,75]^ Furthermore, it can be a disabling factor for adult diseases and acute or chronic diseases.^[77]^ KOK displayed antioxidative activity through various mechanisms, such as the activation of superoxide dismutase or glutathione peroxidase and the reduction of ROS. Based on these mechanisms, KOK exerts significant effects, such as anti-aging, recovery from damage to the central nervous system, increased reproductive capacity, and the alleviation of atopic dermatitis. In other words, KOK could be widely used in chronic, metabolic, and aging diseases in the future.

We analyzed the effects of KOK on the central nervous system and its anticancer activity. KOK affects the nervous system through antioxidant activity, anti-inflammatory activity, the inhibition of acetylcholinesterase, the regulation of acetonic or amino acids, and influencer activity of brain proteins. One study reported that KOK could be applied for the prevention and treatment of Alzheimer’s disease through the PI3K-Akt signaling pathway, the regulation of the actin cytoskeleton pathway, and insulin resistance pathway based on pharmacological analysis.^[78]^ Particularly, 8 studies were related to memory impairment and nerves; therefore, KOK is also effective in neurodegenerative diseases. Subsequently, regarding its anticancer effect, 8 of 10 studies demonstrated the effects through the inhibition of the cell growth rate, the regulation of the cell cycle, and increased percentage of apoptosis in relation to lung cancer. Some studies have reported that KOK inhibits the toxicity of cisplatin, which is used for chemotherapy in western medicine.^[43–45]^ Cisplatin has approximately 40 specific toxicities, including nephrotoxicity, ototoxicity, neurotoxicity, gastrointestinal toxicity, hematologic toxicity, cardiotoxicity, and hepatotoxicity.^[79]^ Of these, nephrotoxicity is predominant, and the overall prevalence of cisplatin‐induced nephrotoxicity in clinical practice has been identified in one-third of the treated patients.^[79,80]^ In addition, concerning the side effects of Western medicine, KOK inhibited the nephrotoxicity of cisplatin,^[46]^ alleviated the toxicity of methotrexate,^[56]^ decreased resistance to *Mycobacterium tuberculosis* when administered in combination with antibiotics,^[64]^ and inhibited liver damage when administered in combination with glutathione.^[67]^ A comprehensive analysis suggested that clinicians can consider the use of KOK as a herbal medicine when considering integrative medicine for patients undergoing cancer treatments, including chemotherapy with cisplatin, in clinical practice.

Additionally, KOK exerts anti-inflammatory and immune effects. The mechanism of its anti-inflammatory effect involves the reduction of cytokines and chemokines, whereas the mechanism of its immune effects involves the reduction of immunoglobulin E, cluster of differentiation 8, and macrophage expression, thereby suggesting that KOK could be applied to inflammatory and immune diseases in the future.

One clinical study and 8 experimental studies reported the toxicity and side effects of KOK. Jang et al^[47]^ reported no toxicity in the liver and kidneys even after relatively prolonged administration (22 days).

In summary, we analyzed 54 studies related to KOK, the majority of which were experimental studies. Its efficacies include antioxidant, anticancer, anti-inflammatory, immune, and growth-promoting activities, in addition to central nervous system, cardiovascular, gastrointestinal, and respiratory effects, without significant toxicity or side effects. However, most of these results were analyzed through experimental studies, thus necessitating additional research to determine the presence of similar effects and safety in humans. Moreover, there have been only 3 clinical studies on KOK, which was reportedly effective in improving health, except for the treatment of pulmonary tuberculosis. Therefore, our review had a limitation in that it was not possible to determine the applicability of the therapeutic effects of KOK analyzed in experimental studies on the human body. Therefore, additional research on the possibility of its clinical application is required.

## 5. Conclusion

KOK can be effective in various diseases through its antioxidant, anticancer, anti-inflammatory, immune, and growth-promoting properties, in addition to the central nervous system, cardiovascular, gastrointestinal, and respiratory effects, without significant toxicity or side effects. Further clinical studies are required in the future to prove its efficacy in clinical practice.

## Acknowledgements

We would like to thank Editage (www.editage.co.kr) for the English language editing.

## Author contributions

**Conceptualization:** Ji-Woo Kim and Ji-Hye Geum.

**Data curation:** Ji-Woo Kim.

**Investigation:** Ji-Woo Kim and Ji-Hye Geum.

**Methodology:** Hyeon-Jun Woo, Yun-Hee Han, and Shin-Hyeok Park.

**Project administration:** Ji-Hye Geum.

**Supervision:** Won-Bae Ha and Jung-Han Lee.

**Validation:** Won-Bae Ha, Hyeon-Jun Woo, Yun-Hee Han, and Shin-Hyeok Park.

**Visualization:** Ji-Woo Kim and Ji-Hye Geum.

**Writing – original draft:** Ji-Woo Kim and Ji-Hye Geum.

**Writing – review and editing:** Ji-Hye Geum, Won-Bae Ha, Hyeon-Jun Woo, Yun-Hee Han, Shin-Hyeok Park, and Jung-Han Lee.
